# The Influence of the Cooling Bores on the Dendritic Structure and Crystal Orientation in Single-Crystalline Cored CMSX-4 Turbine Blades

**DOI:** 10.3390/ma14143966

**Published:** 2021-07-15

**Authors:** Jacek Krawczyk, Włodzimierz Bogdanowicz

**Affiliations:** Institute of Materials Engineering, University of Silesia in Katowice, 1a 75 Pułku Piechoty St., 41-500 Chorzów, Poland; wlodzimierz.bogdanowicz@us.edu.pl

**Keywords:** single-crystalline turbine blades, dendritic structure, crystal orientation, primary arm spacing, local temperature gradient

## Abstract

Single-crystalline cored CMSX-4 blades obtained at a withdrawal rate of 3 mm/min by the vertical Bridgman method were analyzed. The dendritic structure and crystal orientation near the cooling bores of the blades were studied through Scanning Electron Microscopy, the X-ray diffraction measurements of α and β angular components of the primary crystal orientation, and the γ angular component of the secondary crystal orientation. Additionally, the primary arm spacing (PAS) was studied in areas near and far from the cooling bores. It was found that in the area approximately 3–4 mm wide around the cooling bores, changes occurred in the α, β, and γ angles, as well as in the PAS. The PAS determined for the transverse section of the root and the linear primary arm spacing (LPAS) determined for the longitudinal sections, as well as their relationship, have been defined for the areas located near the cooling bores and those at a distance from them. The vertical temperature gradient of 29.5 K/cm was estimated in the root areas located near the cooling bores based on the PAS values. The value of this gradient was significantly higher compared to the growth chamber operating gradient of 16 K/cm. The two-scale analysis applied in this study allowed for the determination of the relationship between the process of dendrite array creation proceeding on a millimeter scale, which is associated with the local changes in crystal orientation near the cooling bores, and that which proceeds on a scale of tens of millimeters, associated with the changes in crystal orientation in the whole blade cast.

## 1. Introduction

The blades operating in the turbine hot section of jet engines must be resistant to, among others, high thermomechanical loads. For this reason, turbine blades are made of heat and creep-resistant materials such as the most recently used single-crystalline CMSX-4 superalloy. Additionally, the blades are produced with cooling channels or bores to reduce their temperature. The single-crystalline (SX) CMSX-4 blades are capable of maintaining high mechanical and fatigue strength, and in particular, high-temperature creep resistance throughout their entire service life [1,2,3,4]. Even a slight increase in the service life of the blades can significantly reduce the operating costs of new generation turbo-jet engines. Understanding the mechanisms that create heterogeneity in the blade structure during crystallization may lead the way to the improvement of the production process, and consequently, to an amelioration of the economic factors.

**Figure 1:**
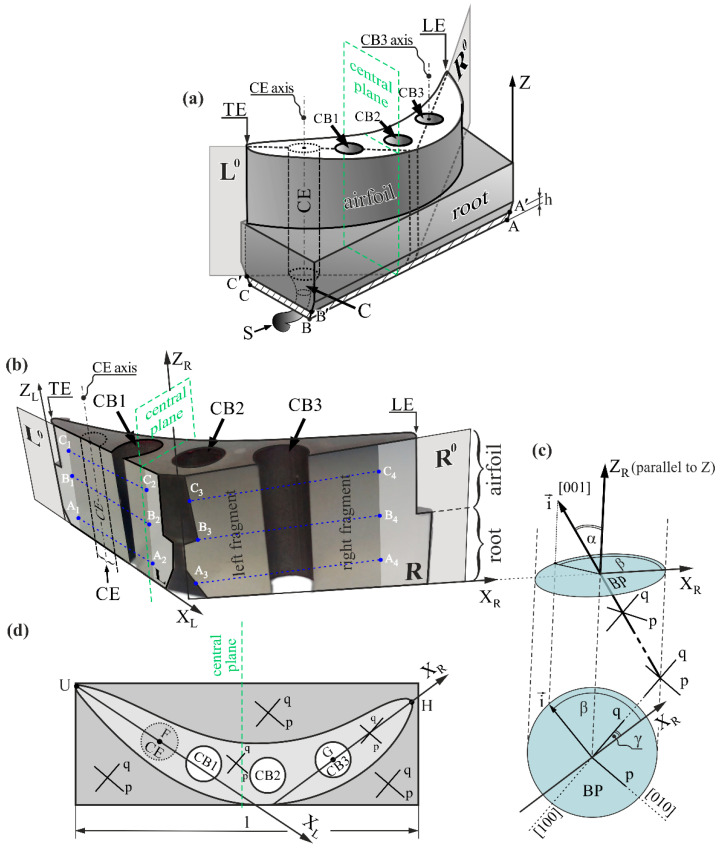
Schema of the blade with the location of the cutting planes L^0^ and R^0^ (**a**); the shape of the sample with two lateral surfaces L and R (**b**); the graphical definition of the primary crystal orientation angular components α and β and the secondary crystal orientation angular component γ (**c**); and top view of the blade with the schema of the secondary dendrite arm orientation (**d**). Z—blade axis, perpendicular to the surface ABC of the root; CB1, CB2, CB3—cooling bores; S—selector; C—continuer; CE—continuer extension area; h—bottom root layer of the unsteady dendrite growth; LE and TE—leading and trailing edge of the airfoil; BP—base plane parallel to the ABC surface and perpendicular to Z; $\vec{i}$—unit vector. The α angle is enlarged for figure clarity; p and q define the alignment of the secondary dendrite arms.

The SX-cored turbine blades are produced by directional dendritic crystallization using the Bridgman technique. The blades with cooling bores are formed using a casting mold with internal ceramic cores [5,6,7,8]. The Bridgman technique allows for the production of blades with the required crystallographic orientation of the [001] type [1], which is almost parallel to the blade axis Z (Figure 1a) and thus with a low α angle characterizing the primary crystal orientation, and a dendritic structure with low spatial heterogeneity. Obtaining blades with high homogeneity of both the dendritic structure and the spatial distribution of the α angle is important because it is related to the structural perfection of the blades and their superior strength and mechanical properties [9,10,11,12]. During its service life, the blade is loaded with a high centrifugal force and this concentration of stress may cause inhomogeneities in the blade. This in turn can cause nucleation and the development of micro-cracks, thus leading to blade failure [3].

The misorientation of the neighboring areas of the dendrite array, described as a local heterogeneity of crystal orientation, can lead to the formation of low-angle boundaries (LABs) that reduce creep resistance and other strength parameters. The reduction can take place in two ways: globally, by the deviation of the mean crystal orientation from the [001] direction, and locally, by local crystal orientation changes causing the formation of LABs [13,14,15]. The dislocation of LABs can cause the strong segregation of the Re alloy additive during high-temperature service [16] as well as the loss of “the solid-solution strengthening effect of Re atoms” [16]. 

The crystal orientation of SX superalloys significantly influences thermal fatigue strength [17]. Additionally, local changes in the crystal orientation of the dendritic structure may affect the corrosion resistance of SX Ni-base superalloys [18]. The inhomogeneity of the dendritic structure may also cause the creation of sliver defects, which would influence the mechanical properties [19].

It is known that any local deviation of the [001] direction from the blade axis Z, described by the α angle (Figure 1c), causes inhomogeneity in the dendritic structure of the blade [1,20,21]. However, full primary orientation is defined by two angles, α and β, which define the arrangement of the primary dendrite arms as a result of their growth in the direction of the unit vector $\vec{i}$. The secondary crystal orientation—described by the γ angle (Figure 1c)—defines the arrangement of the secondary dendrite arms relative to one of the characteristic directions of the blade shape, such as the X_R_ axis presented in Figure 1. This is related to the leading edge (LE) of the airfoil and the axis of one of the cooling bores (CB3 axis). The secondary arms are aligned parallel to the crystallographic directions [100] (q) and [010] (p). The local change of the γ angle indicates the rotation of the neighboring areas of the array of dendrites around the axis of the primary arms (axis of the vector $\vec{i}$). Such a rotation often results in the formation of low angle boundaries (LABs) [22]. Therefore, determining the spatial distribution of γ is very important. The α and β angles may be called the angular components of the primary orientation, and the γ angle can be labeled as the angular component of the secondary orientation. 

In cored turbine blades, the heat transfer rate during directional crystallization and the resulting temperature gradient near the bores may be different from those in the remaining part of the blade. This can significantly change the dendritic array and the primary and secondary crystal orientation. The growth of both the primary and secondary dendrite arms in the [001]-type directions is often changed by the outer walls of the mold, resulting in the bending of arms in the areas near the blade surface [23]. Therefore, it is essential to analyze the α, β, and γ angle distribution in the vicinity of the cooling bores’ side wall, formed by the inner mold surfaces. The variable crystallization conditions of the dendrites’ growth near the cooling bores may also affect the value of the primary arm spacing (PAS) [1], which the creep resistance depends on. In addition, PAS affects the duration of the costly heat treatment of the blades, which is performed at a later stage of production. Therefore, it is essential to analyze the PAS close to the cooling bores and compare it with the PAS measured at a greater distance from them.

The local change in the direction of the primary and secondary arms in the vicinity of the cooling bores may cause the creation of several types of defects during crystallization. A large amount of defects, such as LABs, may reduce the strength and creep resistance of the blade [24,25,26,27,28]. For this reason, it is essential to analyze the dendritic structure by measuring the spatial distribution of the α, β, and γ angles of crystal orientation and the primary arm spacing in the areas located near the cooling bores. So far, no results have been published on the above-mentioned issues.

The aim of the study was to examine the as-cast dendritic structure of single-crystalline cored turbine blades in the vicinity of the cooling bores. The goal was achieved by measuring the changes in the α and β angles of the primary crystal orientation and the γ angle of the secondary crystal orientation, as well as by measuring the changes in the primary arm spacing, depending on the distance from the bore side walls. 

Although cooled blades made of nickel-based superalloys with a dendritic structure have been used for quite some time, some aspects of the formation of structure heterogeneity during crystallization are still not explained. Understanding the mechanisms by which these inhomogeneities are created will indicate ways of reducing or eliminating them in blades of a more complex shape, which can be used in new-generation jet engines. Additionally, extending the service life of the blades can significantly reduce the operating costs of the engines. To understand the mechanisms that create inhomogeneity, it is necessary to study model blades of a simplified shape, which will allow for the separation of the individual mechanisms by which inhomogeneity is created. In the present study, blades with a short airfoil and circular cross-section bores were chosen (Figure 1a). From such model blades, specific samples were prepared with the shape shown in Figure 1b. It was assumed that the study of their lateral surfaces L and R would allow the achievement of the above-mentioned goal. The reasons for selecting the L and R surfaces in this way are presented in the Materials and Methods chapter.

## 2. Materials and Methods

The model blades made of CMSX-4 superalloy were directionally crystallized at a withdrawal rate of 3 mm/min using the vertical Bridgman technique, with the temperature gradient in the growth chamber of G_0_ = 16 K/cm [29]. The airfoil of the obtained model blades was 8 mm long. The blade casts contain three cylindrical cooling bores—CB1, CB2, and CB3 (Figure 1a)—and a spiral selector (S) with a continuer (C), which are asymmetrically positioned in the transverse section of the blade (Figure 1a). The asymmetric position of the selector resulted from the arrangement of the core forming the cooling bore CB2, which had to be connected to the bottom fragment of the casting mold. Locating the selector symmetrically, i.e., under the central plane (Figure 1a), would make the bottom tip of the core of CB2 impossible to fasten to the bottom fragment of the casting mold. The axes and side walls of all cooling bores and axis Z of the blade were perpendicular to the base ABC plane. Additionally, the leading (LE) and trailing (TE) edges of the airfoil were also perpendicular to the base ABC plane of the root. The cylindrical shape bounded by the projection, orthogonal to the ABC plane, of the continuer’s perimeter into the blade was called the continuer extension (CE) area. The side surfaces and axis of the CE were perpendicular to the ABC plane (Figure 1a,b). The industrial ALD Vacuum Technologies furnace used at the Research and Development Laboratory for Aerospace Materials in Rzeszów University of Technology, Rzeszów, Poland, was employed to produce the blades. 

In the first step of the blade sample preparation, the bottom root layer h, with a thickness of about 5 mm (Figure 1a), in which dendritic crystallization could occur under unsteady conditions [30], was cut-off and excluded from the study. In the second step, the metallographic section of the bottom surface AʹBʹCʹ of the root (Figure 1a) was prepared. The lengths of the AʹBʹ and BʹCʹ edges were 55 mm and 12 mm, respectively. In the third step, a sample of the shape presented in Figure 1b was prepared by cutting off the blade’s fragment along the planes L^0^ and R^0^, which were parallel to the blade axis Z (Figure 1a). The L^0^ plane intersects the CE area and consists of its axis (Figure 1a,b,d). Additionally, the L^0^ plane includes both the CE axis and trailing edge (TE) of the airfoil, as indicated in Figure 1d by points F and U, while the R^0^ plane includes both the CB3 axis and the leading edge (LE) of the airfoil, as indicated in Figure 1d by the points G and H. The samples obtained, as described above, have lateral surfaces L and R, with the shape presented in Figure 1b. The microsections of the L and R surfaces were prepared using the standard metallographic procedure intended for Ni-based superalloys [31]. Fragments of the L and R surfaces placed near the CB1 and CB2 and marked in dark gray in Figure 1b were studied. The fragment of the L surface, placed to the left of the CB1, covers the cross-section area of the CE. It was assumed that the CE area, where the dendrites grow directly from the selector and continuer, consists of the lowest number of misoriented areas, which then allowed for its cross-section area to be used as reference to those located near the CB1.

The JSM-6480 JEOL SEM microscope (JEOL Ltd., Tokyo, Japan) was used to visualize the dendritic structure of the fragments of L and R and the AʹBʹCʹ surfaces analyzed by back-scattered electron (BSE) imaging. The SEM macro images of the studied surface fragments were created by collecting several separate micro images. The values of the α, β, and γ angles were measured by the Ω-scan method at points forming lines perpendicular to the CB1 and CB3 side walls, using a dedicated EFG Freiberg Instruments X-ray diffractometer (Freiberg Instruments, Freiberg, Germany) [32]. The mean error of the α, β, and γ angle measurements was 0.006°. The measurement lines were indicated as A_1_-A_2_, B_1_-B_2_, and C_1_-C_2_ for fragments of the L surface, as well as A_3_-A_4_, B_3_-B_4_, and C_3_-C_4_ for fragments of the R surface (Figure 1b). Exemplary left and right fragments of the R surface are presented in Figure 1b. Lines A_1_-A_2_ and A_3_-A_4_ were positioned at the bottom part of the root, where the dendrites began to grow in steady conditions [30]. Lines B_1_-B_2_ and B_3_-B_4_ were positioned in the connection area between the root and the airfoil. Lines C_1_-C_2_ and C_3_-C_4_ were located halfway up the airfoil (Figure 1b). The measurement point step was 0.5 mm for all lines. The collimated incident beam of the characteristic CuKα radiation covered an area of 0.8 mm in diameter on the analyzed surface. The unit vector $\vec{i}$ (Figure 1c), arranged parallel to the [001] direction, defines the growth direction of the primary dendrite arms [1,30].

## 3. Results and Discussion

**Figure 2:**
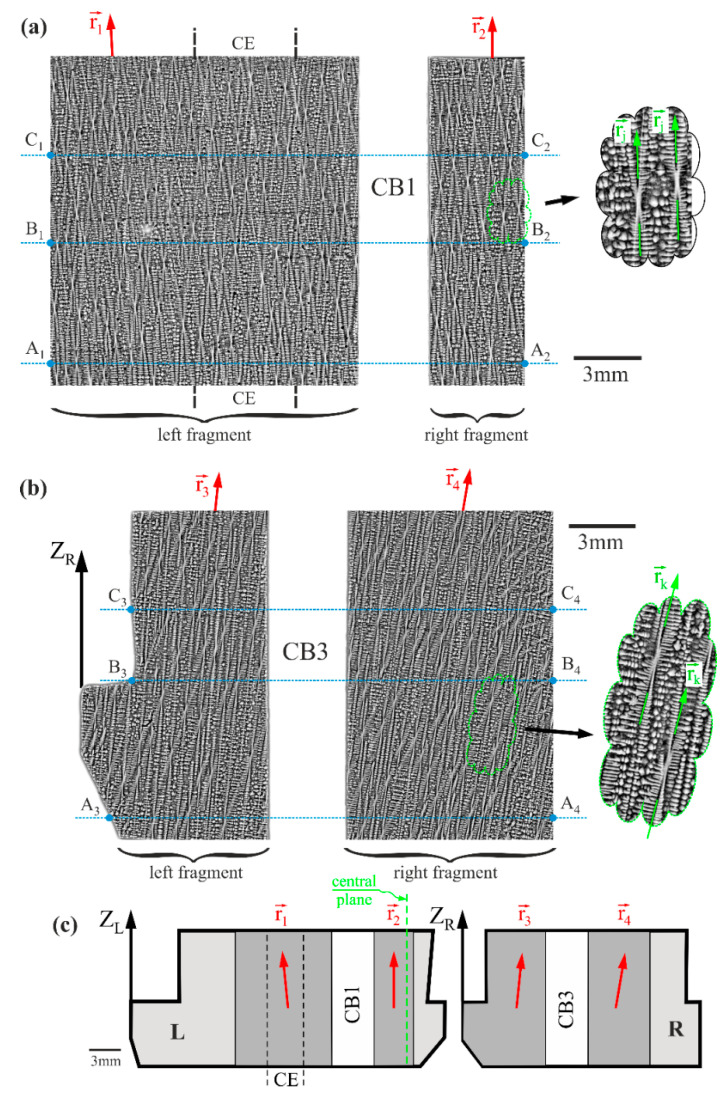
SEM macro images of the dendritic structure visualized on the left and right fragments of surface L (**a**) and surface R (**b**), as well as the schema of the “fanning effect” of primary dendrite arms (**c**). BSE technique. CB1, CB3—cooling bores, CE—continuer extension area. The inserts on the right show exemplary hourglass-like shapes that visualize single dendrites on the L and R surfaces. $\vec{r_{1}}-\vec{r_{4}}$ and $\vec{r_{j}}$, $\vec{r_{k}}$—unit vectors that define the growth direction of primary dendrite arms and axes of hourglass-like shapes, respectively.

Figure 2a,b present exemplary SEM macro images of the dendritic structure, visualized on the left and right fragments of surface L and surface R, respectively. The dendritic structure typical for single-crystalline casts made of nickel-based superalloys can be observed. The inserts on the right side show exemplary hour-like shapes that visualize single dendrites on the fragments of the L and R surfaces. These shapes were created by oblique cuts through the dendrites by the planes of the R and L surfaces, which means that the primary dendrite arms grew non-precisely parallel to those planes. The hourglass shapes visualized on both fragments of the L surface are symmetrical to their axes (axes $\vec{r_{j}}$, Figure 2a—insert). Additionally, on the left fragment of the L surface, the axes represented by the unit vector $\vec{r_{1}}$ are inclined to the left. This means that the primary dendrite arms grew inclined to the left, as indicated by the vector $\vec{r_{1}}$. In contrast, on the right fragment of surface L, those arms grew approximately vertical, that is, parallel to the blade axis Z (Figure 1a), as indicated by the vector $\vec{r_{2}}$. On both fragments of the R surface, the hourglass-like shapes are distorted and asymmetric relative to their axes (axes $\vec{r_{k}}$, Figure 2b—insert), which are inclined to the right (Figure 2b). This means that the primary dendrite arms are inclined to the right as indicated by the vectors $\vec{r_{3}}$ and $\vec{r_{4}}$. Additionally, the dendrite inclination, represented by the inclination of vector $\vec{r_{4}}$, is higher for the right fragment of the R surface.

The inclination to the left of the primary dendrite arms on the left fragment of the L surface (Figure 2a) and to the right on the left and right fragment of the R surface (Figure 2b), as well as their parallel orientation to the *Z* axis on the right fragment of the L surface located near the central plane of the blade root (Figure 1d), are consistent with the “fanning effect” described in [20,33]. A schema of the effect is presented in Figure 2c. This effect is global—it applies to the entire blade casting. The scale of the effect is comparable to the dimension of the root’s edge A’B’, which is tens of millimeters. 

**Figure 3:**
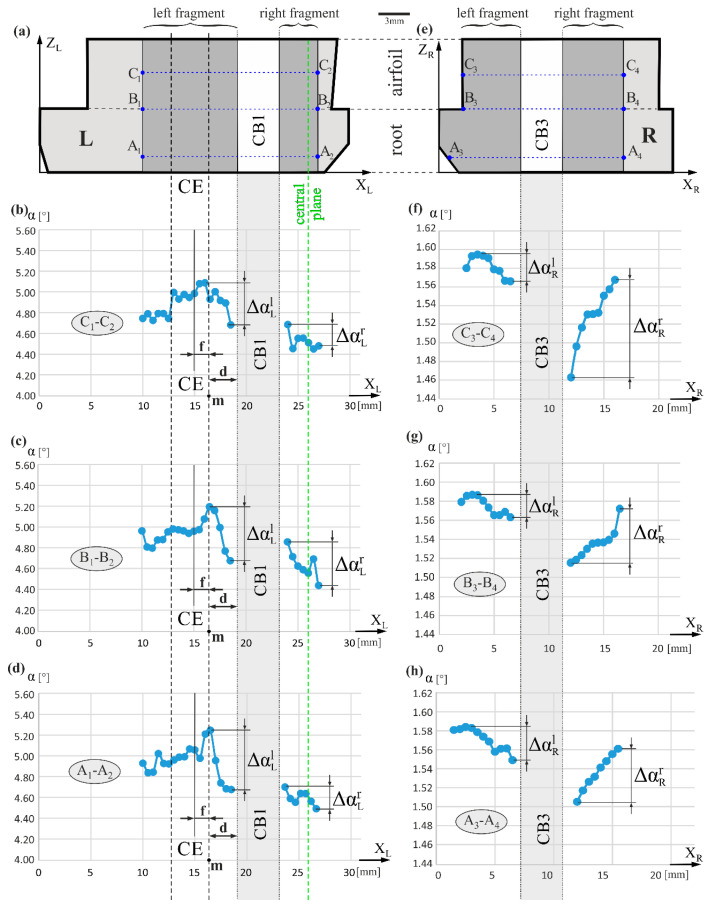
Schema of the arrangement of the α angle measurement lines (**a**,**e**), and the α values distribution on the fragments of the L (**b**–**d**) and R (**f**–**h**) surfaces along these lines in the vicinity of the cooling bores (CB1 and CB3). The labels of the measurement axes are replaced by parallel axes X_L_ and X_R_ for simplicity of analysis.

Figure 3 shows the arrangement of the α measurement lines and the distribution of the α values along the X_L_ and X_R_ axes near CB1 and CB3. The distributions are similar to those presented in [34] because the data were obtained from blades produced in the same series, i.e., under the same crystallization conditions. Additionally, the samples had the same shape and were prepared in the same way. Because all the measurement lines are parallel to the X_L_ and X_R_ axes, the axes of the lines are marked as X_L_ and X_R_ for description simplification. The α value is one of the angular components of the dendrite orientation, whose primary arms grow in the direction indicated in Figure 1c by the unit vector $\vec{i}$. An analysis of α changes on the left fragment of the L surface and on the left and right fragments of the R surface indicate that near both bores, the value of α generally decreases against the background of slight stochastic fluctuations. For the left fragment of the L surface, the reduction of α, marked in Figure 3b–d as $\Delta \alpha_{L}^{l}$ for lines located both in the root (A_1_-A_2_) and in the airfoil (C_1_-C_2_), as well as at the level of the root–airfoil connection (B_1_-B_2_), varies from 0.4° to 0.6°. These reductions occur in area *d*, about 3 mm in width. For the left part of surface R, a reduction of this type, marked as $\Delta \alpha_{R}^{l}$, ranges from 0.02° to 0.04° for all measurement lines A_3_-A_4_, B_3_-B_4_, C_3_-C_4_. However, for the right fragment of surface R, a reduction of this type, marked as $\Delta \alpha_{R}^{r}$, varies on average from 0.06° to 0.1° for all measurement lines. However, for the right fragment of surface L, when reducing the distance to CB1, the α value increases. This increase $\Delta \alpha_{L}^{r}$ ranges from 0.3° to 0.4° for all measurement lines. A reduction of the α near the cooling bores is likely due to the “force-directing” of growing primary dendrite arms parallel to the increased vertical temperature gradient near the side surface of CB1 and CB3. In turn, the increase in temperature gradient is related to heat transfer through the cores of the casting mold forming CB1 and CB3. In contrast, increasing the α on the right part of the L surface ($\Delta \alpha_{L}^{r}$) may be related to a more global “fanning effect” (Figure 2c), resulting in the primary arms growing even more parallel to the Z_L_ (Z) axis as they come closer to the central plane of the root (Figure 1b and Figure 3a–d). The “fanning effect” is defined as global if it occurs over the entire analyzed area of the blade. 

For the left fragment of the L surface, the decrease of α caused by the global “fanning effect” is added to the more local “force-directing” effect near CB1. However, for the right fragment of the L surface, the “force-directing” effect is unnoticeable against the background of the prevailing “fanning effect”. For the left fragment of the R surface, these effects are similar and work against each other, resulting in $\Delta \alpha_{R}^{l}$ changes being small compared to larger $\Delta \alpha_{R}^{r}$ changes caused by the consistent influence of both effects. 

On the left fragment of the L surface, the additional *f* area can be distinguished (Figure 3b–d), which is part of the CE area. In the *f* area, the α value increases when reducing the distance from CB1 and is higher than the α in the rest of the CE. The effect may be related to the curvature of the crystallization front at the border of CE, described in [13,35].

**Figure 4:**
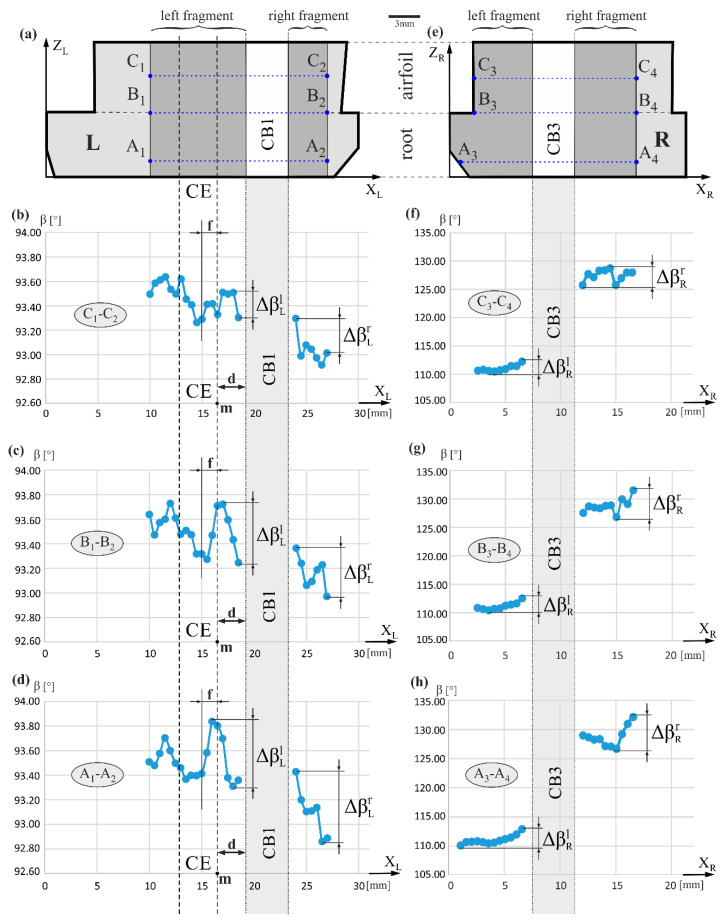
Schema of the arrangement of the β angle measurement lines (**a**,**e**), and the distribution of the β values on the fragments of the L (**b**–**d**) and R (**f**–**h**) surfaces along these lines in the vicinity of the cooling bores (CB1 and CB3). The labels of the measurement axes are replaced by parallel axes X_L_ and X_R_ for simplicity of analysis.

Figure 4 shows the arrangement of the β measurement lines and the distribution of the β values along lines A_1_-A_2_, B_1_-B_2_, C_1_-C_2_ and lines A_3_-A_4_, B_3_-B_4_, C_3_-C_4_, on axes marked as X_L_ and X_R_, respectively. The value of the β angle on the left and right fragments of the L surface, specified for all three lines A_1_-A_2_, B_1_-B_2_, and C_1_-C_2_, are close to 90°. The β angle defines the rotation of the projection of the unit vector $\vec{i}$ [001] on the BP around the X_L_ or X_R_ axes. Figure 1c shows an example of the β angle for the rotation around the X_R_ axis for the R surface. On the left fragment of the L surface, an area denoted as *d* and about 3 mm wide may be distinguished; in this area, the β value decreases by $\Delta \beta_{L}^{l}$ in the range of 0.2°–0.6° as the distance from CB1 decreases. However, in the alternative area denoted as *f*, which is part of the CE, the β value generally increases in a similar range for all measurement lines. On the right fragment of the L surface, for all measurement lines, the β value generally increases by $\Delta \beta_{L}^{r}$ in the range of 0.4°–0.6° when reducing the distance from CB1. This trend occurs against the background of rather large fluctuations. When comparing the β value on the right and left sides of CB1, it can be noticed that β generally decreases as the X_L_ coordinate increases. This means that the decrease is global in nature and probably related to the “fanning effect”. On the left fragment of the R surface, the β value increases by $\Delta \beta_{R}^{l}$ in the range of 2.5°–3.0° for all measurement lines. On the right fragment of the R surface, the β value increases by $\Delta \beta_{R}^{r}$ in the range of 4.0°–7.0° for all measurement lines. This trend occurs against the background of a large fluctuation. For both fragments of the R surface (Figure 4f–h), the β changes near the CB3 are of the order of a few degrees and are much higher than the β changes determined for both fragments of the L surface. For both fragments of the R surface, there is an increase in the β as the X_R_ coordinate increases. This effect is also likely to be related to a global “fanning effect”.

The difference in the β value on the right side of CB3 compared with that on the left side of CB3 is approximately 15–20 degrees. These values are much higher than the changes in the α component. The β angle is independent of the α because the α and β are two independent components of the unit vector $\vec{i}$ (Figure 1b). A change in the β value indicates the rotation of the growth direction of the primary dendrites arms (i.e., the direction of the unit vector $\vec{i}$) around the Z_R_ and Z axes. Such a rotation in the range of a few or several dozen degrees is not directly related to the change of the α angle.

**Figure 5:**
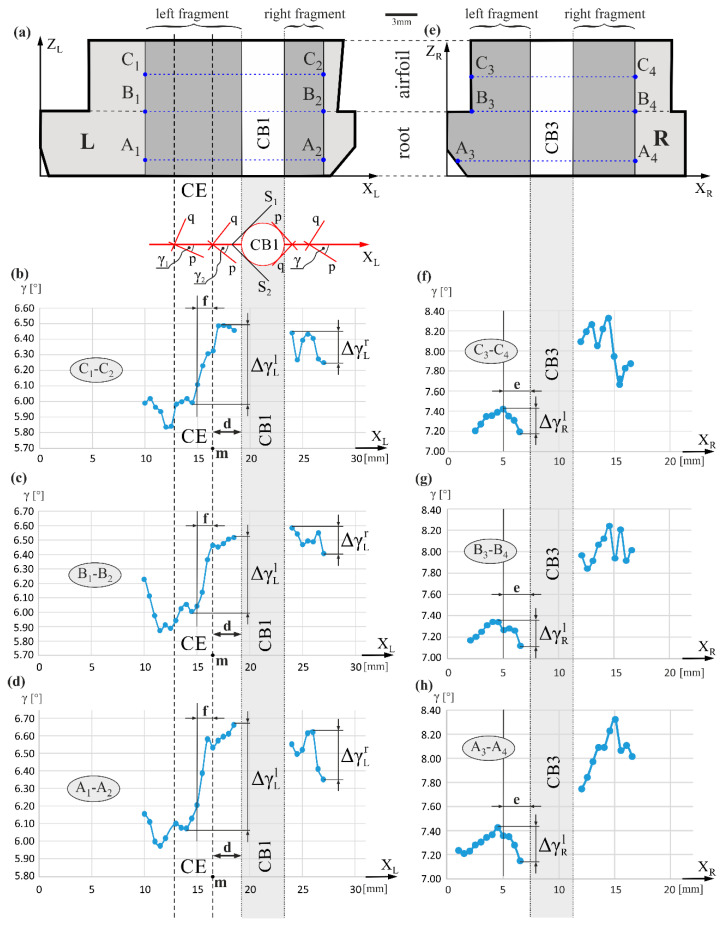
Schema of the arrangement of the γ angle measurement lines (**a**,**e**), and the γ value distribution on the fragments of the L (**b**–**d**) and R (**f**–**h**) surfaces along these lines in the vicinity of the cooling bores (CB1 and CB3). The insert below (**a**) represents the top view of the blade. The labels of the measurement axes are replaced by parallel axes X_L_ and X_R_ for simplicity of analysis.

Figure 5 presents the arrangement of the γ angle measurement lines A_1_-A_2_, B_1_-B_2_, C_1_-C_2_ and A_3_-A_4_, B_3_-B_4_, C_3_-C_4_, and distributions of the γ values along these lines, on axes marked as X_L_ and X_R_, respectively. The γ angle defines the [100] or [010] direction arrangement relative to the X_L_ or X_R_ axis. It can be assumed that the γ angle defines the arrangement of the secondary dendrite arms relative to the X_L_ or X_R_ axis. For the relationships shown in Figure 5, the γ value determines the angle between the X_L_ or X_R_ axis and the angularly closest secondary arm direction. An example related to the X_R_ axis is provided in Figure 1c, where the γ angle is the angle between X_R_ and direction *q*—[100]. The value of this angle varies between 7.1° and 8.3°, specified for all fragments of the measurement lines on the R surface. In the *e* area of the left fragment of the R surface, γ decreases by $\Delta \gamma_{R}^{l}$ in the range of 0.2°–0.3°, along with a decrease in the distance to the side surface of the CB3. This trend occurs both in the root (A_1_-A_2_) and in the airfoil (C_3_-C_4_), and on the boundary between them (B_3_-B_4_). On the other hand, for a portion of the right fragment of the R surface, the γ(X_R_) relationship is complex and is probably related to the existence of multiple low-angle boundaries (LABs) that were created by the rotation of the subgrains, mainly around the axes of the primary dendrite arms. The presence of this type of LABs on the right fragment of the R surface has been confirmed in [30,34]. For the left fragment of the L surface, the γ angle increases by $\Delta \gamma_{L}^{l}$ in the range of 0.5°–0.6°, as the distance to the side surface of CB1 decreases. For the right fragment of the L surface, the changes $\Delta \gamma_{L}^{r}$ of the γ angle with the value of about 0.2°–0.3° have a similar character but against the background of higher fluctuations. The γ value in area *f*, about 1 mm wide and located on the left fragment of the L surface, increases particularly quickly. This involves the rotation of the secondary arms *p* and *q*. As for the reduced distance to the side surface of CB1, the secondary arm *p* (insert below Figure 5a) is arranged so that its direction gradually approaches the direction of the tangent lines S_1_ and S_2_ on the side surface of CB1. The trend of γ changes on the right fragment of the R surface varies. It can be related to the occurrence of numerous LABs with misorientation angles of several degrees [30] over the entire R surface, which significantly distorts the growth of the secondary dendrites arms.

**Figure 6:**
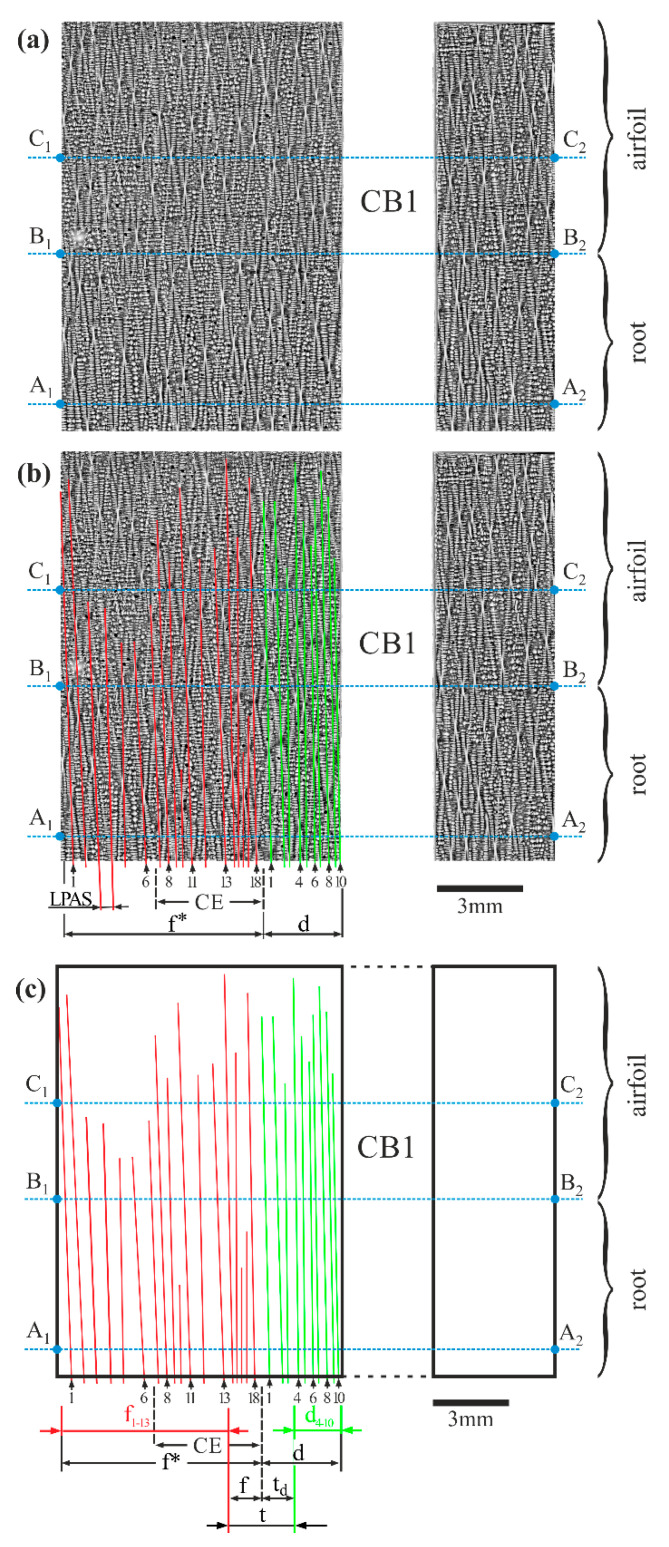
The dendritic structure of the fragments of the L surface (**a**); the structure with numbered lines marking primary dendrite arms on the left of CB1 with marked exemplary LPAS distance (**b**); and schema of the primary dendrite arm arrangement (**c**).

Local changes of the dendrite array near the cooling bores are weakly visible on the SEM macro images of the longitudinal microsection (Figure 2a,b and Figure 6a). However, an attempt was made to define the interdendritic distance in area *d*, which is located near CB1, and in area f*, which is located at some distance from CB1 (Figure 6b), by measuring the linear primary arm spacing (LPAS), also defined previously in [30,36]. The LPAS is the average distance between the primary arms of the dendrites visualized on the longitudinal surface section and measured along the X_L_ axis. One exemplary LPAS distance is marked in Figure 6b. The LPAS values were determined based on the schema of the dendrite arrangement presented in Figure 6c. The almost vertical dendrite arms are presented as numbered lines, marked with green and red, for area *d* and area f*, respectively. As may be seen in Figure 6b, the f* area includes the CE area. The lines in Figure 6c, representing the individual primary dendrite arms, were defined using the criteria described in [36]. The lines of the primary dendrite arms could only be clearly traced for the root area of the blade. In the airfoil, above line B_1_-B_2_, and particularly above line C_1_-C_2_, the growth directions of the primary arms changed so that the continuation of the lines traced for the microstructure image of the root could not be determined. The analysis of the schema presented in Figure 6c shows that generally, the LPAS is smaller in area *d* than that in f*. However, near the boundary of *d* and f*, that is, the boundary between area *d* and CE, a transition area *t* can be distinguished; here, the above-mentioned relationship is distorted. This area, consisting of the sub-areas *t_d_* and *f*, was not considered for the LPAS calculations. In sub-area *t_d_*, which includes three dendrites, the LPAS is higher than that found in area *d*_4–10_ (Figure 6c), which includes seven dendrites. In sub-area *f*, which includes five dendrites, the LPAS is lower than in sub-area *f*_1–13_, which includes thirteen dendrites. As the width and position on the X_L_ axis of area *f* in Figure 6 are comparable to the width and position of area *f* in Figure 3, Figure 4 and Figure 5, the notation is the same in all figures. Generally, the area of changes in LPAS, which includes the *d* and *f* areas, is about 4 mm wide. The LPAS value specified for area *d*_4–10_ was 0.36 ± 0.07 mm (for 7 dendrites), and for the *f*_1–13_ area, the value was 0.67 ± 0.2 mm (for 13 dendrites). The rather large errors in determining the LPAS were related to the small number of dendrites in each area, which only allowed for the determination of the difference in the LPAS values of the two areas. To more precisely determine the interdendritic distance, one of the standard methods based on a transverse-sectional analysis should be used. However, it should be emphasized that an LPAS analysis using the longitudinal section is especially useful for visualizing the changes in interdendritic distances of the primary arms in small areas. The standard method of measuring the primary arm spacing using the transverse section of SX casts does not offer such a possibility.

**Figure 7:**
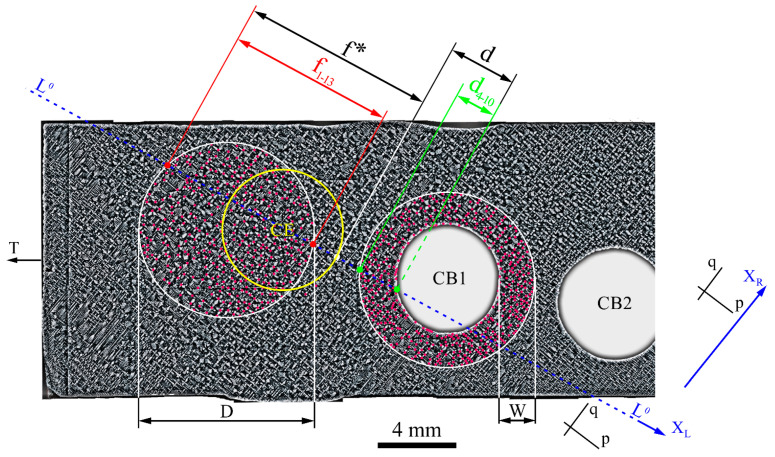
The dendritic structure obtained from the transverse section of the root fragment with marked *f*_1–13_ and *d*_4–10_ areas of PAS analysis. L^0^—trace of the longitudinal plane marked in Figure 1a, along which the dendritic structure of *f*_1–13_ and *d*_4–10_ areas on the left fragment of surface L, visible in Figure 6, was visualized.

In order to compare the PAS between the areas directly adjacent to CB1 and those located far away from it and devoid of its influence, the area *d*_4–10_ and the area d_1–13_ were selected for analysis (Figure 6c). Thus, the transition areas *f* and *t_d_* (Figure 6c) were excluded from the PAS study. The dendritic structure of areas *f*_1–13_ and *d*_4–10_ may be additionally visualized in the transverse section of the root. The transformed SEM macro image of the corresponding fragment of the bottom root surface AʹBʹCʹ (Figure 1a) is presented in Figure 7. The transformation is based on a mirror reflection of the structure image in relation to the T axis. This allowed the structure to be presented as shown in Figure 1d, which represents a top view of the structure. The primary arm spacing (PAS) was determined both in the ring-shaped area *d*_4–10_ around CB1 at a width of W = 1.8 mm, and in the circular area *f*_1–13_ with a diameter of D = 9.4 mm, which includes the fragment of CE. The W and D values were chosen so that the widths of the areas *d*_4–10_ and *f*_1–13_ marked in Figure 7 would be equal to those marked in Figure 6c. It has been assumed that the PAS, defined in the *d*_4–10_ ring-shaped area, corresponds to the LPAS in the *d*_4–10_ area shown in Figure 6c; on the other hand, the PAS, defined in the circular area of a diameter D, was assumed to correspond to the LPAS in the area *f*_1–13_ marked in Figure 6c. The “dendrites array method”, based on the relationship of a square array of points [37], was chosen for PAS determination. Calculations were made using the equation: PAS = c · (A/n)^0.5^, where *n* is the number of dendrite cores marked in Figure 7 by red points, A is the area of the surface defined for counting the dendrites when measuring PAS, and c = 1 for a “square array” according to [38]. The calculated PAS values for the *d*_4–10_ ring-shaped area around CB1 and the circular area *f*_1–13_ were as follows: PAS_4–10_ = 0.28 ± 0.03 mm; PAS_1–13_ = 0.38 ± 0.05 mm. The PAS_4–10_ value in the area *d*_4–10_ and the PAS_1–13_ value in the area *f*_1–13_ were determined with the use of 635 and 470 dendrite cores, respectively.

The standard method for PAS determination, which is the “dendrites array method”, is based on the analysis of transverse sections normal to the [001] direction. The PAS value in the *d*_4–10_ area is lower in relation to area *f*_1–13_. The relation between LPAS defined for the *d*_4–10_ and *f*_1–13_ areas of the left fragment of the transverse L surface (Figure 6c) are similar—LPAS is lower in *d*_4–10_ in comparison to the LPAS in *f*_1–13_ area. There is a correlation between the changes in PAS and LPAS, although the changes in the LPAS value are overstated. The reason may be that some dendrites presented in the transverse section areas are invisible in the longitudinal section areas. Lower PAS values in the area *d*_4–10_ near CB1 indicate that the vertical temperature gradient near CB1 is elevated. 

Knowing the PAS in the *f*_1–13_ area—hereinafter referred to as PAS_1–13_, and the PAS near the cooling bore in the *d*_4–10_ area—hereinafter referred to as PAS_4–10_, the value of the temperature gradient near CB1 can be determined. The PAS values can also be calculated from the well-known equation presented, for example, in [39] and used in simplified form (1), which takes into account only the dendrite growth rate *v* and the temperature gradient *G* in the areas of dendrite growth.

(1) $$\mathrm{PAS}=B\cdot v^{-\frac{1}{4}}\cdot G^{-\frac{1}{2}},$$

where *B* is constant.

Since the micro-section of the A’B’C’ plane (Figure 1a) was located in the area of steady dendrite growth (the bottom layer of the root, which could be created under unsteady growth conditions, was cut off), it can be assumed that the dendrite growth rate in the *d*_4–10_ and *f*_1–13_ areas was the same and equal to the withdrawal rate from the high-temperature zone of the growth chamber in the Bridgman furnace, i.e., *v*_0_ = 3 mm/min. It was also assumed that parameter *B* is constant for both areas. In addition, it was assumed that the temperature gradient *G* in the *f*_1–13_ area covering CE is equal to the nominal furnace gradient $G_{0}$ ($G_{1-13}=G_{0}=16$ K/cm).

From the above assumptions, it follows that:

(2) $${\mathrm{PAS}}_{1-13}=B\cdot v_{0}^{-\frac{1}{4}}\cdot G_{0}^{-\frac{1}{2}}.$$

From Equation (2), parameter *B* can be determined:

(3) $$B=\frac{{\mathrm{PAS}}_{1-13}}{v_{0}^{-\frac{1}{4}}\cdot G_{0}^{-\frac{1}{2}}}.$$

For the area *d*_4–10_ near the CB1:

(4) $${\mathrm{PAS}}_{4-10}=B\cdot v_{0}^{-\frac{1}{4}}\cdot G_{4-10}^{-\frac{1}{2}},$$

where *G*_4–10_ is the vertical temperature gradient near the CB1.

By using the *B* from Equation (3) in Equation (4), it follows that:

(5) $$PAS_{4-10}=\frac{PAS_{1-13}}{G_{0}^{-\frac{1}{2}}}\cdot G_{4-10}^{-\frac{1}{2}}.$$

From Equation (5), the following calculations can be made:

(6) $$\frac{1}{\sqrt{G_{4-10}}}=\frac{PAS_{4-10}}{PAS_{1-13}}\cdot \frac{1}{\sqrt{G_{0}}}$$

(7) $$\mathrm{and}\;G_{4-10}=G_{0}{\left(\frac{PAS_{1-13}}{PAS_{4-10}}\right)}^{2}.$$

Using Equation (7) and the known values for *G*_0_, PAS_1–13_, and PAS_4–10_, the *G*_4–10_ value was calculated, which is 29.5 K/cm.

The *G*_4–10_ value may be overestimated because it was assumed in Equation (1) that *B* is a constant. A more detailed analysis should consider that the chemical composition of the *d*_4–10_ area will be different from that in the area *f*_1–13_.

The first-time application of the Ω-scan method with the use of the EFG diffractometer, preparation of specific samples with surfaces specially selected for tests, and using the LAS parameter for analysis, allowed for the formulation of the following conclusions, which have not previously been presented in any available publication.

## 4. Conclusions

Local changes in the α and β angles of the primary crystal orientation and the γ angle of the secondary crystal orientation occur in areas 3–4 mm in width located near the side wall of the blade cooling bores. These changes are caused by the different alignment of both the primary and secondary dendrite arms, depending on their distance from the cooling bores. Close to the cooling bores, the dendrite arms are more parallel to the side walls of the bores. These local changes are overlapped by changes caused by the “fanning effect”, which are more global in nature and concern the entire blade casting. Additionally, near the side wall of cooling bores, the primary arm spacing (PAS) decreases to 0.28 mm in relation to the areas at a distance from the bores, where the PAS is about 0.38 mm. This is caused by an increase in the vertical temperature gradient near the cooling bores. It was estimated for the blade root that the vertical temperature gradient near the side wall of the cooling bores was 29.5 K/cm, contrary to the growth chamber operating gradient, which was 16 K/cm. The linear primary arm spacing (LPAS) method is very useful for visualizing the changes in the interdendritic distances of the primary arms in small areas. The standard method of measuring the primary arm spacing using the transverse section of SX casts does not offer such a possibility. The description of the dendritic array of cored turbine blades requires analysis at least on two scales: local, in the order of millimeters—related to the local changes in crystal orientation near the cooling bores, and global, in the order of tens of millimeters—related to the changes in crystal orientation in the whole blade cast, i.e., related to the “fanning effect”.

## Data Availability

Not applicable.
